# The first comprehensive genomic characterization of rectal squamous cell carcinoma

**DOI:** 10.1007/s00535-022-01937-w

**Published:** 2022-11-11

**Authors:** Christoforos Astaras, Claudio De Vito, Prasad Chaskar, Aurelie Bornand, Kaouthar Khanfir, Amedeo Sciarra, Igor Letovanec, Claudia Corro, Pierre-Yves Dietrich, Petros Tsantoulis, Thibaud Koessler

**Affiliations:** 1 Medical Oncology Department, Geneva University Hospitals, 4 rue Gabrielle-Perret-Gentil, 1205 Geneva, Switzerland; 2 Pathology Department, Geneva University Hospitals, Geneva, Switzerland; 3 Radiation Oncology Department, Valais Hospital, Sion, Switzerland; 4 Histopathology, Central Institute, Valais Hospital, Sion, Switzerland; 5 Swiss Cancer Center Léman, Lausanne, Geneva Switzerland; 6 Translational Research Center in Onco-Hematology, Department of Medicine, Faculty of Medicine, University of Geneva, 1205 Geneva, Switzerland

**Keywords:** Rectal squamous cell carcinoma, Anal squamous cell carcinoma, Rectal adenocarcinoma, Next-generation sequencing, Human Papilloma Virus

## Abstract

**Background:**

Rectal cancers represent 35% of colorectal cancers; 90% are adenocarcinomas, while squamous cell carcinoma accounts for 0.3% of them. Given its rarity, little is known concerning its pathogenesis, molecular profile and therapeutic management. The current treatment trend is to treat rectal squamous cell carcinoma by analogy to anal squamous cell carcinoma with definitive chemo-radiotherapy, setting aside surgery in case of local recurrence.

**Methods:**

We performed an in-depth genomic analysis (next-generation sequencing, copy number variation, and human papilloma virus characterization) on 10 rectal squamous cell carcinoma samples and compared them in silico to those of anal squamous cell carcinoma and rectal adenocarcinoma.

**Results:**

Rectal squamous cell carcinoma shows 100% HPV positivity. It has a mutational (*PIK3CA*, *PTEN*, *TP53*, *ATM*, *BCL6*, *SOX2*) and copy number variation profile (3p, 10p, 10q, 16q deletion and 1q, 3q, 5p, 8q, 20p gain) similar to anal squamous cell carcinoma. PI3K/Akt/mTOR is the most commonly affected signaling pathway similarly to anal squamous cell carcinoma. Most commonly gained or lost genes seen in rectal adenocarcinoma (*FLT3*, *CDX2*, *GNAS*, *BCL2*, *SMAD4*, *MALT1*) are not found in rectal squamous cell carcinoma.

**Conclusion:**

This study presents the first comprehensive genomic characterization of rectal squamous cell carcinoma. We confirm the existence of this rare histology and its molecular similarity with anal squamous cell carcinoma. This molecular proximity confirms the adequacy of therapeutic management based on histology and not localization, suggesting that rectal squamous cell carcinoma should be treated like anal squamous cell carcinoma and not as a rectal adenocarcinoma.

**Supplementary Information:**

The online version contains supplementary material available at 10.1007/s00535-022-01937-w.

## Introduction

Rectal cancer (RC) represents ∼35% of colorectal cancers (CRC) [1] representing 125,000 new cases per year in Europe. Its treatment is associated with significant morbidity and mortality [2]. More than 90% of rectal tumors are adenocarcinomas (ADC) [31], rectal squamous cell carcinoma (rSCC) is very rare and accounts for 0.2% to 0.4% of all RC. The localization of SCC in the rest of the colon is even rarer [4, 5]. Although the role of human papillomavirus (HPV) in the tumorigenesis of anal SCC (aSCC) is well documented, association between HPV and colorectal SCC is still controversial and not proved [6–9]. On the other hand, Coghill et al. in a large retrospective study show an increased risk to develop rSCC among patient with advanced immunosuppression [10]. In terms of prognosis, staged matched, rSCC seems to have to a poorer prognosis compared to rectal adenocarcinoma (rADC) [11]. According to Dyson et al. [12], rSCC have a less favorable prognosis than SCC of the rest of the colon. Prognostic factors associated with better survival are: early stage, younger age, female sex, African/American race and use of radiotherapy (RT).

Given the rarity of rSCC, some authors question its existence. To diagnose primary rSCC, the fulfillment of 4 (William’s) criteria is required [13]: (1) No continuity between the tumor and the anal squamous epithelium or the gynecological tract; (2) Absence of a SCC in another primary site; (3) Absence of squamous-lined fistula in the context of inflammatory bowel disease; (4) Finally histological confirmation.

As far as immuno-histochemistry is concerned, rSCC and rADC express cytokeratin CAM5.2, unlike aSCC, suggesting a common cell of origin for these two rectal cancer subtypes [4], while P63 are frequently expressed by rSCC and aSCC and CK20 in rADC. 34bE12 seems more frequently expressed by rSCC and aSCC than ADC.

There is no clear consensus about which TNM classification (AJCC-anus or AJCC-rectum) should be used for rSCC staging. A large population-based study of 2′881 rSCC patients concluded that AJCC-anus staging system offers a better prognostic discrimination compared to AJCC-rectum and should therefore by preferred to predict patients’ survival [14].

While rADC are treated with radiotherapy (RT) combined with chemotherapy of 5-fluorouracil (5-FU) followed by surgery [1, 15], aSCC are treated with definitive radiation therapy with concomitant chemotherapy of mitomycin C (MMC) and 5-FU, setting aside surgery in case of local recurrence [15–19]; for rSCC, no clear recommendations exist. In the past, surgery was the standard of care, based on retrospective and observational studies [20]. More recently, small series have hinted that definitive concomitant chemo-radiotherapy (CRT), lead to high rates of partial or complete pathological response as well as organ preservation [20]. Our study aims at clarifying the role of HPV in rSCC as well as comparing rSCC, aSCC and rADC molecular profile.

## Materials and methods

### Sample selection

We searched our pathology reports databases for patients with rSCC, using the keywords “squamous cell carcinoma” and “rectum” and selected those who meet the inclusion criteria. We also used our SNOMED (Systematized Nomenclature of Medicine) International Code coding system, looking for the codes “squamous cell carcinoma” and “rectum” associated with our reports. Only tissue from patients with rSCC that meets the following 4 strict diagnostic criteria has been used: (1) No continuity between the tumor and the anal squamous epithelium or the gynecological tract, (2) Absence of a SCC in another primary site, (3) Absence of squamous-lined fistula in the context of inflammatory bowel disease, (4) Finally histological confirmation of rSCC. All the cases of rSCC biopsy, in whom the complementary work-up (pelvic MRI, ano-rectoscopy, colonoscopy) shows an anal origin (or extension) of the gynecological tract, or another primary tumor location (non-rectal) have been excluded. The study is authorized by the competent Ethics Committee of Geneva (Project-ID: 2021-00,149).

### Patient cohort

We identified nine patients at HUG (Geneva University Hospitals) and another three cases were selected from the cantonal hospital of Sion (2 institutions study). These two institutions count about an average of 70 new rectal cancer cases per year, meaning approximately 2100 patients in the last 30 years (period during which pathology reports databases have been searched in our study). In other words, our 10 rSCC cases correspond to 0, 4% of all rectal cancers of our 2 centers, a percentage that is in absolute adequacy with rSCC’s prevalence in the literature.

Tissue sample selection was performed during the first half of 2021. After careful histological review, two cases were dropped—one had mixed adeno-squamous histology and the other was a poorly differentiated adenocarcinoma. One patient (clinical case 1) was initially diagnosed with a concomitant squamous esophageal carcinoma in addition to his rSCC. To understand if both tumors were related (exclusion criteria), we carried out an HPV profiling, which showed positivity for the rectal tumor and negativity for the esophageal tumor, speaking in favor of 2 unrelated tumors.

The 10 selected patients (Table 1) were profiled by NGS sequencing, CNV analysis and HPV typing. Both tumor and non-tumor tissues from the 10 selected patients are used in our assays. In 3 out of 10 patient cases (clinical cases 5, 6, and 10), molecular analyses were carried out on post-CRT samples, because of their higher percentage of tumor cells compared to tissue biopsies taken on pre-CRT.

**Table 1:** An overview of the patients’ cohort

	Diagnosis	Treatment	Outcome
Clinical case1 81 y.o (M)	04.2016 Well to moderately differentiated, non-keratinizing, invasive, squamous cell carcinoma (2 cm) of the lower rectum (not classified) Tissue: initial biopsy	Not treated, due to poor general condition and comorbidities	Died a few months later
Clinical case 2 63 y.o. (F)	02.2016 Well to moderately differentiated, partly keratinizing invasive squamous cell carcinoma of the middle rectum, classified cT4 N2 M1 (hepatic metastases) Tissue: pre-RCT biopsy	04-06.2016: 4 × TCF 08-09.2016: rectal radio (60 Gy)-chemotherapy (5-FU + MC) 02-05.2017: FOLFIRI 07-09.2017: nivolumab	Died 11.2017
Clinical case 3 83 y.o. (F)	11.2006 Moderately differentiated, keratinizing and ulcerated, squamous cell carcinoma (3 × 1 cm) of the lower rectum (not classified) Tissue: initial biopsy	No treatment administrated (rapid progression and poor general condition in the context of gastric adenocarcinoma with peritoneal carcinosis)	Died 01.2007
Clinical case 4 48 y.o. (F)	06.2001 Poorly differentiated squamous cell carcinoma of the lower rectum, classified as uT3 N1 (para-rectal) M0 Tissue: pre-RCT biopsy	06-08.2001: rectal definitive radio (65 Gy) -chemotherapy (5-FU + MC), with complete tumoral response	Long-term remission
Clinical case 5 57 y.o. (F)	11.2002 Moderately differentiated squamous cell carcinoma of the lower rectum, classified as uT3 N0 M0, with hepatic metastatic relapse (04.2003) and local rectal relapse (08.2003) Tissue: pre-RCT biopsy	11.2002–01.2003: rectal definitive radio (60 Gy)-chemotherapy (5-FU + MC), with complete local tumoral response 05.2003: left hepatectomy 06-08.2003: 5-FU + CBDCA 09.2003: low abdomino-peritoneal amputation	No recent information available
Clinical case 6 85 y.o. (F)	06.2007 Squamous cell carcinoma (basaloid variant) ulcerated of the lower rectum, classified as uT3 N1 (para-rectal) M0, with local relapse (02.2010) Tissue: post-RCT surgery (relapse)	06-08.2001: rectal definitive radio (65 Gy)-chemotherapy (MC) with complete tumoral response 05.2010: hemostatic radiotherapy (6 Gy) and surgery of relapse	No recent information available
Clinical case 7 55 y.o. (F)	03.2013 Poorly differentiated, keratinizing, squamous cell carcinoma of the upper rectum, perforated, classified cT4 N1 (external iliac) M0, with vaginal relapse Tissue: post-CRT surgery	04-05.2013: rectal radiotherapy (40 Gy)-patient refused chemotherapy, with partial response 07.2013: abdomino-peritoneal amputation (radiotherapy not feasible, chemotherapy and pelvic exenteration refused by patient)	Died a few months later
Clinical case 8 66 y.o. (M)	11.2011 Invasive squamous cell carcinoma of the middle third of the rectum classified at least cT3 N1 M0 Tissue: pre-CRT biopsy	12.2011–02.2012: rectal definitive radio (59.4 Gy) -chemotherapy (5-FU + MC), with complete tumoral response	Disease free. Last FU 17.12.2020
Clinical case 9 62 y.o. (M)	02.2007 Invasive squamous cell carcinoma and severe dysplasia of carcinoma in situ of the lower rectum classified at least cT1 N1 M0 Tissue: pre-CRT biopsy	04-06.2007: rectal definitive radio (64.4 Gy)–chemotherapy (5-FU + MC), with complete tumoral response	Disease free. Last FU 19.08.2021
Clinical case 10 63 y.o. (F)	01.2010 Moderately differentiated, non-keratinizing, squamous cell carcinoma of the lower rectum (not classified), with local relapse 11.2012 08.2014: probable relapse (peritoneal carcinosis) 10.2015: lung metastasis and ileus Tissue: post-CRT surgery	03-04.2010: rectal definitive radio (59.4 Gy)–chemotherapy (5-FU + MC), with complete tumoral response 03.04.2013: surgery of the relapse (abdomino-peritoneal amputation): poorly differentiated squamous cell carcinoma with superficial ulceration	Died 12.2015

*5-FU* 5-flururacil,* MC* mitomycine, *CBDCA* carboplatin, *FU* follow-up, *M* Male, *F* Female, *CRT* chemo-radiotherapy

### DNA sequencing and CNV analysis

Genomic DNA extraction and purification using the QIAamp DNA FFPE (Fixed-Formalin, Paraffin-embedded) tumor tissue Kit (cat. 56,404; QIAGEN, Hilden, Germany) and copy number profiling and quantification, using the OncoScan Assay kit (cat. 902,695; ThermoFisher Scientific) were performed following manufacturer’s instructions, as previously described [21]. For NGS sequencing, libraries of a custom 462-gene panel (SureSelect-HS library, Agilent) were built from genomic DNA. Paired-end sequencing, 2 × 150 nt, has been performed on a NextSeq500 sequencer (Illumina) as previously described [22]. The size of our custom NGS panel is > 1Mbp.

Copy number variation was performed, using the OncoScan Assay kit (cat. 902,695; ThermoFisher Scientific) following manufacturer’s instructions, as previously described [21]. Data were analyzed using OncoScan Console and Chromosome Analysis Suite (CHAS) software.

CNV segments were classified into four categories: “gain’’, when there are one or two extra copies with respect to the diploid state; “amplification’’, in case of a gain of five or more copies; “loss’’, when the number of copies is lower than the normal number (two in a human genome); and loss of heterozygosity (LOH), when there is a loss of the maternal or paternal allele without any loss of copies.

The Cancer Gene Census, COSMIC (Catalogue of Somatic Mutations In Cancer), CIViC (Clinical Interpretations of Variants in Cancer), OncoKb (PMID: 28,890,946) were used for variant interpretation and classification according to international guidelines (PMID: 25,741,868, PMID: 27,993,330).

### HPV detection

DNA extracted from fixed material was of sufficient quality to perform PCR-Blot analysis (DNA control and HPV positivity). Analysis for HPV virus DNA was realized by polymerase chain reaction (PCR) amplification of the region conserved L1 and hybridization of the PCR product on blot, making it possible to identify high-risk HPV types (16,18,31,33,35,39,45,51,52,56,58,59,68a), probably high risk (26,53,66,70,73,82), low risk (6,11,40,42,43,44,54,61), and HPV of uncharacterized pathogenicity (62,67,83,89).

For HPV genotyping, the Inno-LiPA HPV Genotyping Extra II (cat.81534, Fujirebio) was used according to the manufacturer’s instructions. We proceeded to a PCR analysis using a kit that can detect the presence of HPV and then hybridization to determine the HPV subtype.

### Data validation

We hypothesized that comprehensive mutation profiling of a cohort of rSCC tumors, could assist in defining the genomic landscape of this rare cancer. We compared our data with public rADC databases from TCGA (The Cancer Genome Atlas Program) [22–26]. Concerning aSCC, there is no public database including a complete molecular characterization of this tumoral entity. In this context, we used genomic profile published in the scientific literature for aSCC in pre- and post-CRT for local and metastatic disease [25, 27, 28]**.**

## Results

### Molecular analysis

HPV Assessment: All patients (10/10) were positive for high risk HPV16, one of them was positive for high risk HPV16 as well as high risk HPV18.

**Fig. 1:**
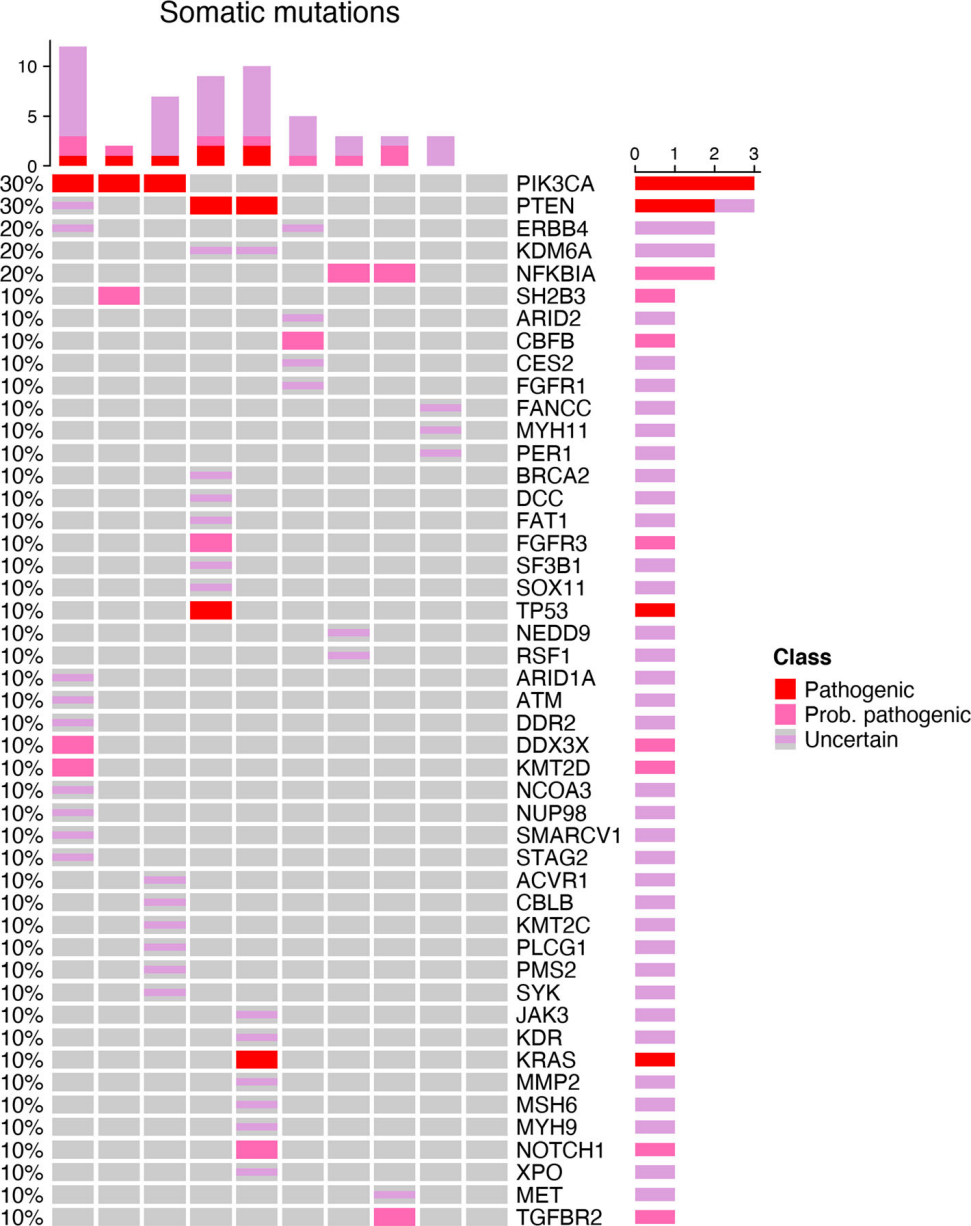
Frequency and pathogenicity of somatic mutations-10 rSCC clinical cases

NGS analysis: We identified between 0 and 13 mutations per sample. The most frequent pathogenic variant was found in PIK3CA and PTEN genes (Fig. 1). Tumor Mutation Burden (number of non-synonymous mutations per mega-base) was generally low and heterogeneous (range, 0 and 9 mutations per Mb).

**Fig. 2:**
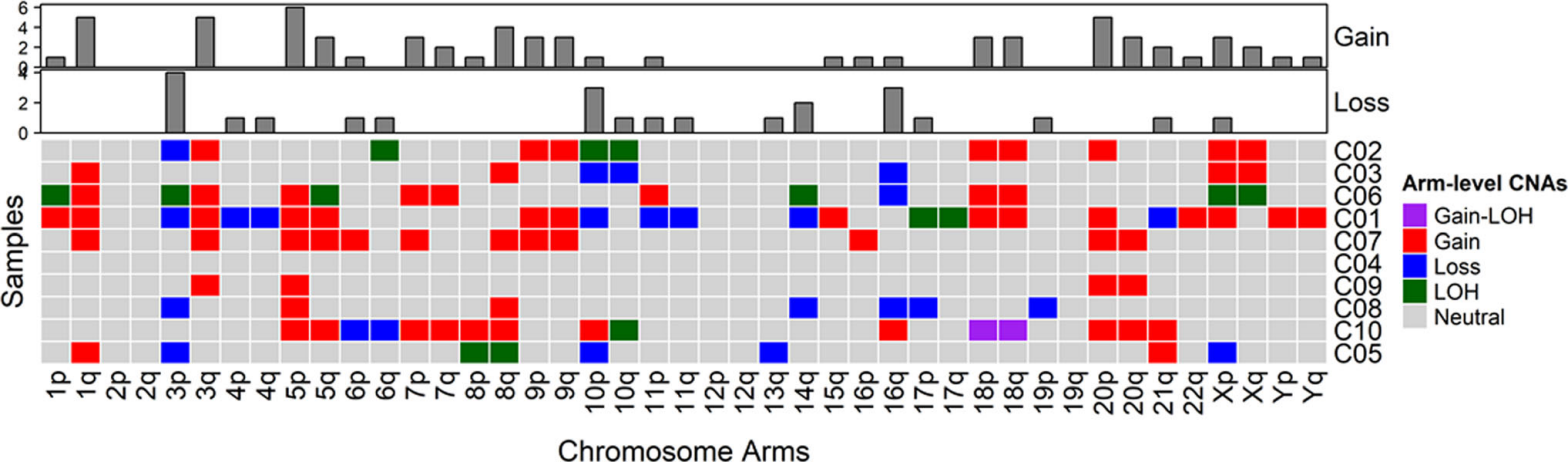
Landscape of somatic CNV of our 10 rSCC clinical cases

*Oncoscan analysis*: In Fig. 2, we show an overview of the landscape of somatic copy number variations (CNV) in the 10 cases. The most frequently deleted regions in rSCC are in chromosomal arms 3p, 10p, 10q, and 16q-the 10q23 region containing *PTEN* gene (heterozygous and homozygous loss have been found). The five regions most commonly harboring gains are in chromosomal arms 1q, 3q, 5p, 8q and20p-the 3q26 and 3q27 regions containing *PIK3CA*, *SOX2*, *BCL6* genes.

**Fig. 3:**
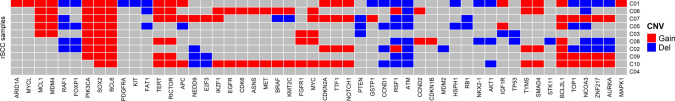
Top 50 most altered genes-our 10 rSCC clinical cases

The 50 most altered (gains or losses) genes found by Oncoscan are depicted in Fig. 3. The genes most frequently gained were PIK3CA (8/10), *SOX2* (9/10), *BCL6* (9/10), *TERT* (6/10) and *BCL2L1* (7/10). The genes most commonly lost were *ATM* (7/10), *PTEN* (3/10), *RSF1* (6/10) and *RAF1* (5/10). An overview of the 10 karyotypes performed (via Oncoscan) as well as the most notable alterations of our 10 rSCC cases (Supplementary Fig. 2) is depicted in as an attachment.

Mutation and CNV–pathway analysis: The combined analysis of somatic mutations and CNV shows that recurrent alterations (*PIK3CA, PTEN*) of the PI3K/AKT/mTOR pathway are the most frequent in rSCC.

### In silico* comparison between rSCC and rADC*

Using the TCGA database, we extracted the most frequent copy number variations (CNV), genes in rADC. The most frequent gains concern *FLT3*,* CDX2*,* GNAS*,* and BCL2L1* genes whereas the most prevalent losses are observed in *BCL2*,* SMAD4*,* MALT1* genes.

**Fig. 4:**
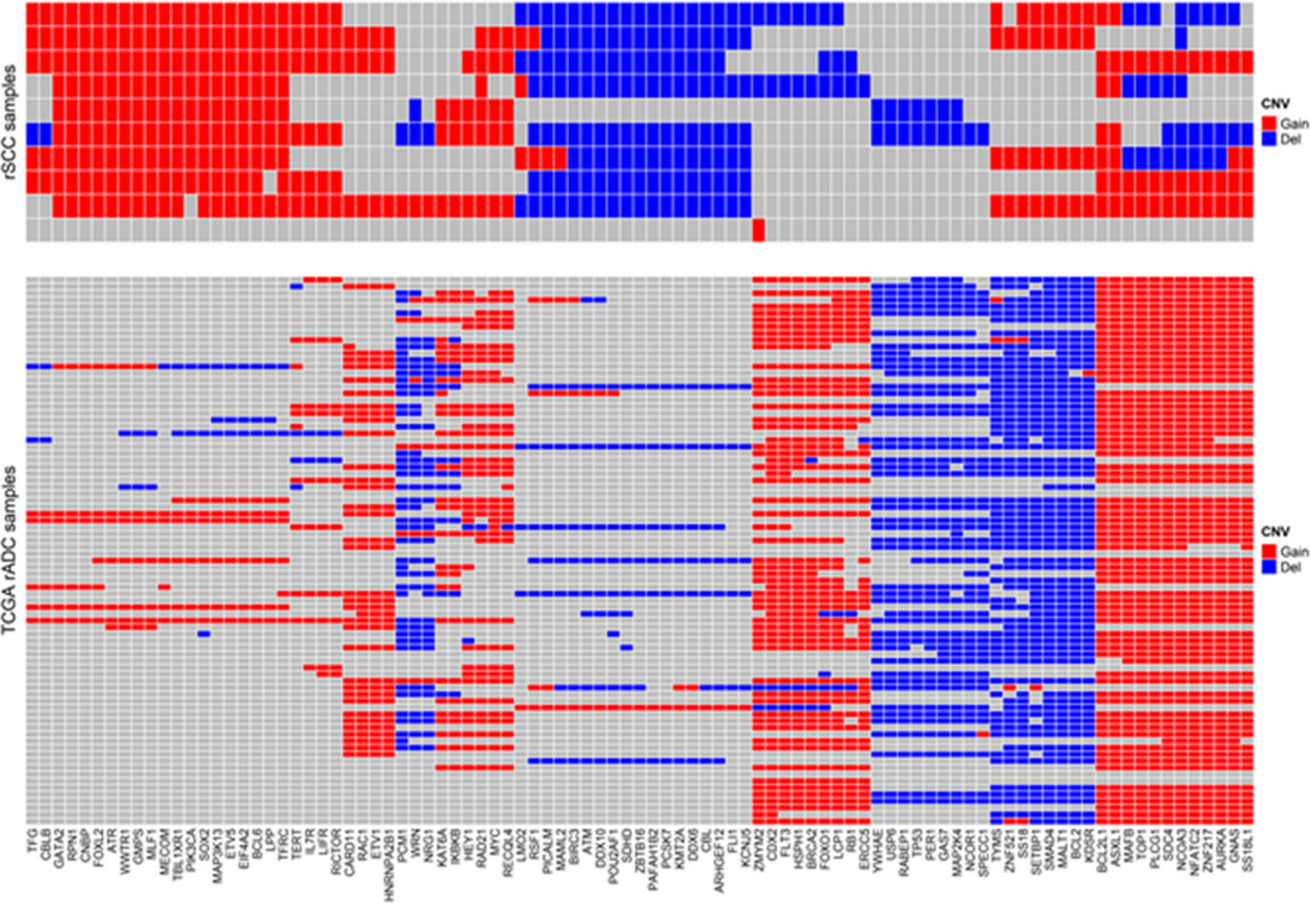
Comparison of top 100 most altered genes between rADC and 10 rSCC cases

**Fig.5:**
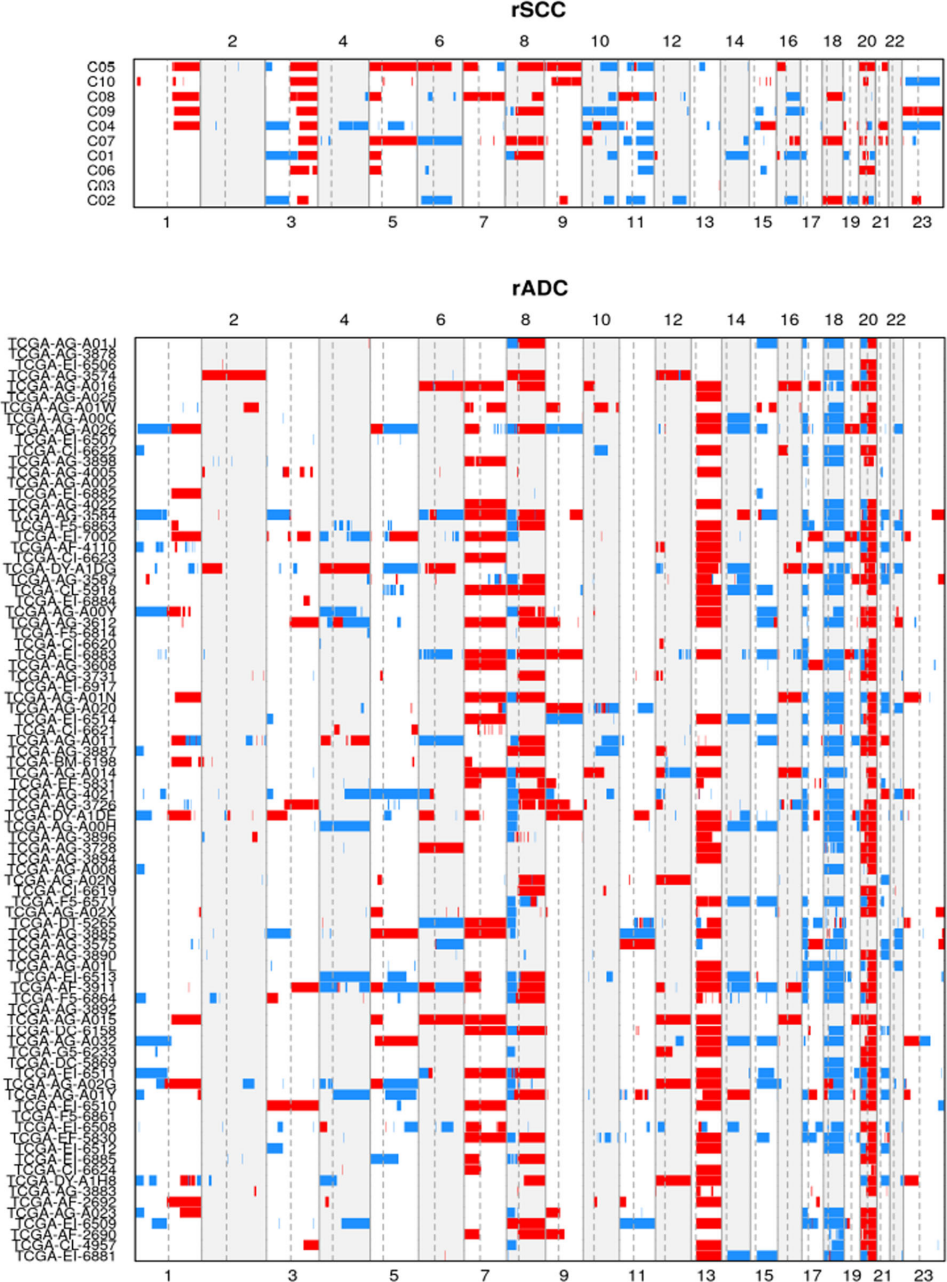
Whole genome comparison between rSCC and rADC datasets copy number alteration. (The mean copy number per genome segment is plotted with gains colored in red, and losses in blue)

To visually compare rSCC to rADC (Fig. 4), we plotted the most frequently lost or gained genes in those 2 tumor entities. A whole genome comparison between rSCC and rADC datasets copy number alteration is depicted in Fig. 5. The CNV mean values per genome segment for both rSCC and rADC have been plotted with the function plot aberration from the R package copy number [29]. In both figures, rSCC’s common CNV are rarely present in the rADC’s samples and inversely, the most common CNV present in rADC samples are not present in rSCC.

As no constituted databases exist for aSCC, we extracted data from the literature. Several series having performed comprehensive genomic analysis in aSCC tumors, show prevalent mutations of *PIK3CA*, *FBXW7*, *TP53*, *PTEN* and *ATM* genes, similar to those found in our 10 rSCC cases. To compare frequently muted genes between rSCC and rADC, we used public databases of rADC mutational profile. The most frequently mutated genes are *KRAS*, *TP53*, *APC*, and* FBXW7*, genes and at a lesser frequency *SMAD4*, *BRAF, CTNNB1*, and *ERBB2* [30].

## Discussion

This study is the first in-depth molecular characterization of squamous cell carcinoma of the rectum with HPV characterization, gene mutation profiling and CNV in 10 patients diagnosed with rSCC according William’s criteria [13]. Our data prove that this entity exists and shows high similarity with aSCC and supports the current approach to treat rSCC similarly to aSCC—with definite CRT setting aside surgery in case of local recurrence.

HPV is detected in all our cases, with high risk HPV16 being the most prevalent genotype (10/10) and high risk HPV18 being also present in 1 case (1/10). According to the literature ^[31,32]^, 88% of aSCC are positive for HPV, with HPV16 being the most frequent HPV subtype (75–80% of all aSCC) followed by HPV18 (3,5–7%). Furthermore, 2 of the 10 cases harbored *TP53* mutations. In different series (25), presence of *TP53* mutation in aSCC is likely to be associated with HPV-negative tumors and confers poor prognosis as well as tumor relapse. It is licit to now consider HPV as a risk factor for rSCC like it is for aSCC and contrary to rADC [33, 34]. This result gives an additional argument for treating rSCC like aSCC.

NGS analysis showed that *PIK3CA* and *PTEN* are the most frequent (30%) mutated genes in rSCC followed by *ERBB4*, *KDM6A* and *NFKBIA* with 20%. Recent targeted sequencing studies of aSCC showed that *PIK3CA* is frequently mutated with the same frequency (30%) than in our study [23, 27]. Interestingly, *PIK3CA* is also significantly mutated in other HPV-associated cancers, such as head and neck [28, 35, 36] or cervical cancers [37]. *PTEN* is also mutated in 30% of our cases, highlighting the major role of the PI3K/Akt/mTOR pathway in rSCC carcinogenesis, similarly to aSCC [25]. *PIK3CA* and *PTEN* are also associated to response to therapies targeting this pathway in other squamous cell or HPV-associated carcinomas [24]. Cacheux et al^23^ suggest that *PIK3CA* mutations might play a major role in HPV-related aSCC, including anal carcinogenesis, especially in mechanisms of resistance to CRT.

Among the most frequently mutated genes, mutation in *PIK3CA*, *PTEN*, and *ATM* is considered possibly actionable. They can be targeted by specific tyrosine kinase inhibitors, such as alpelisib for PIK3CA [38], AKT inhibitors like capivasertib for PTEN [39] and ATR inhibitors like elimusertib for ATM [40]. We showed that rSCC mutational profile has almost no overlapping with rADC one. Based on our findings, we do not believe routine testing for mutations in *KRAS*,* NRAS*, and *BRAF* is likely to yield significant results for rSCC. Although we cannot exclude the presence of rare mutation in these genes, it is unlikely that those mutations would influence clinical decision-making in the management of metastatic rSCC.

Among most frequent copy number variation, regions in rSCC are three deleted regions in chromosomal arms 3p, 10p, and 16q and five gained regions in chromosomal arms 1q, 3q, 5p, 8q and 20p. Region 3p loss and region 3q gain are dominant features of the squamous cancer clusters and are present in cervical and anal squamous cell carcinomas, HPV-positive head and neck squamous cell carcinomas as well as in esophageal squamous cell carcinomas [27, 41]. Gain of 5p is the most frequent karyotypic change in gynecological cervical cancer, which is also closely related to HPV [42]. Gain in 8q chromosomal arm and especially in 8q24 region harboring amplifications in *CSMD3*, *MYC* and *ASAP1* genes, has been described in different type of cancers [43, 44]. Concerning chromosome 20p11 gains (C20orf3 gene), they are associated with liver-specific metastasis in patients with CRC [45]

Our study has several possible biases. First, the number of cases recruited is limited (10 patients) due to the rarity of the tumor entity studied. This element could question the representativeness of the molecular profile of these cases compared to the true molecular profile of rSCCs. It is certainly true for any alteration with a frequency below 10%. However, we believe we have captured the most frequent ones. Furthermore, the genomic proximity to aSCC—a tumor that is genomically well characterized—makes us confident that we have found the most relevant alterations.

Another important issue is that for 3 of our cases, molecular analyses were carried out on post-CRT samples, because of a high percentage of tumor cells on the samples. We cannot exclude that the CRT caused some genomic alterations (DNA double-strand breaks induced by RT). Even if this is the case, we have no way to identify those molecular differences because a comparison with the primary untreated tumor tissue is not made (not possible given the low presence of tumor cells in the last one). It is worth mentioning that in a study analyzing with whole-exome sequencing primary and recurrent (after CRT) aSCCs, tumors harbored the same mutations and mutational burden [27].

All those aforementioned findings support three important conclusions: firstly, rSCC exists as an entity and is defined by very specific clinical criteria. Previous studies found in the literature concerning rSCC, contain data from retrospective series and registry analyses. The quality of those data is highly debatable, as we are not sure that authors respected all of the 4 William’s criteria (it is not clearly mentioned in all of the studies). For instance, some of them do not explain if one of the principal requirements is fulfilled; if the epicenter of the tumor is well (at least 2 cm) above the dentate line or if tumor arise in the anus and extend up into the rectum. In our study, we have done our best to be as selective as possible and be sure that all the diagnostic criteria are met.

Second, rSCC molecular profile (gene mutated, copy number variation) shows similarity with aSCC and different from rADC. Finally and most importantly, this work confirms that rSCC should be treated like aSCC and not as a rADC. It is clear that a large, multicenter, formal, prospective clinical trial with rSCC cases would be of great interest. Larger cohorts using a variety of genomic approaches, including methylation as well as transcriptomic, epigenetic and proteomic analyses are needed to further characterize this entity. They may also provide additional power to detect differences in mutational patterns that reflect the influence of genomic exposure to DNA/damaging agents, in relation to patients having been treated by CRT or not. Furthermore, identifying predictive biomarkers of CRT response could allow clinicians to escalate therapy or incorporate novel agents for tumors harboring genomic predictors of increased recurrence risk (such as *PIK3CA* mutations) and could be a challenge in rSCC as well as in other tumors for which CRT is used in a curative approach. Our work highlights the importance of genomic characterization of rare cancers to help guiding clinical management.

## Supplementary Information

Below is the link to the electronic supplementary material.

Supplementary file1 (DOCX 332 KB)

## Abbreviations

ADC: Adenocarcinoma
aSCC: Anal squamous cell carcinoma
CBDCA: Carboplatin
CDDP: Cisplatin
CEA: Carcinoembryonic antigen
CNA: Copy number alterations
CNV: Copy number variations
CRC: Colorectal cancer
CRT: Chemo-radiotherapy
DNA: Deoxyribonucleic acid
FDG-PET/CT: Fluorodeoxyglucose-positron emission tomography/computed tomography
ERUS: Endorectal ultrasound
ESMO: European Society of Medical Oncology
HPV: Human papillomavirus
LOH: Loss of heterozygosity
MMC: Mitomycin
MRI: Magnetic resonance imaging
NCCN: National comprehensive cancer network
NGS: Next-generation sequencing
OS: Overall survival
PCR: Polymerase chain reaction
RC: Rectal cancer
RNA: Ribonucleic acid
RT: Radiotherapy
rADC: Rectal adenocarcinoma
rSCC: Rectal squamous cell carcinoma
SCC: Squamous cell carcinoma
TNM: Tumor-node metastasis
5-FU: 5-Fluorouracil
