# Supplementary material for: The first comprehensive genomic characterization of rectal squamous cell carcinoma
Source: J Gastroenterol. 2022 Nov 11;58(2):125–34. doi: 10.1007/s00535-022-01937-w (PMC9876866; doi:10.1007/s00535-022-01937-w)
Supplement: Supplementary file 1 — Supplementary file1 (DOCX 332 KB) [file 535_2022_1937_MOESM1_ESM.docx]

**Supplementary Figure 1 :** Karyotypes and Copy Number Analysis of our 10 rSCC patients

**Clinical case 1
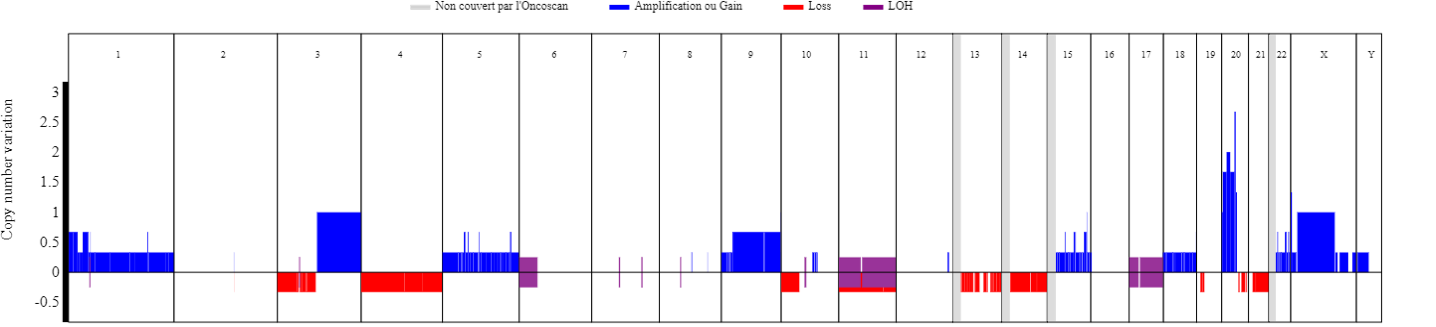
**

**Alterations concerning whole arms or chromosomes:**

Gain: 1q, 1p, 3q, 5q, 5p, 9q, 9p, 15q, 18p, 18q, 20p, 22q, Xq, Xp, Yq, Yp.

LOH: 11q, 17q, 17p

Loss: 3p, 4q, 4p, 10p, 11q, 11p, 14q, 21q

**Notable alterations**

| Genomic Position | Cytoband | Size(Kbp) | Number of copies | Type | Genes of interest |
| --- | --- | --- | --- | --- | --- |
| 20:29'938'061-32'877'766 | 20q11.21 - 20q11.22 | 2'939 | 4.67 | Gain (focal) | BCL2L1 |
| 3:93'517'442-197'852'564 | 3q11.1 - 3q29 | 104'335 | 3 | Gain | PIK3CA |
| 13:28'610'182-33'850'251 | 13q12.2 - 13q13.1 | 5'240 | 1.67 | Loss heterozygous (focal) | BRCA2 |
| 17:25'326'940-80'263'427 | 17q11.1 - 17q25.3 | 54'936 |  | LOH | BRCA1 |

**Clinical case 2**
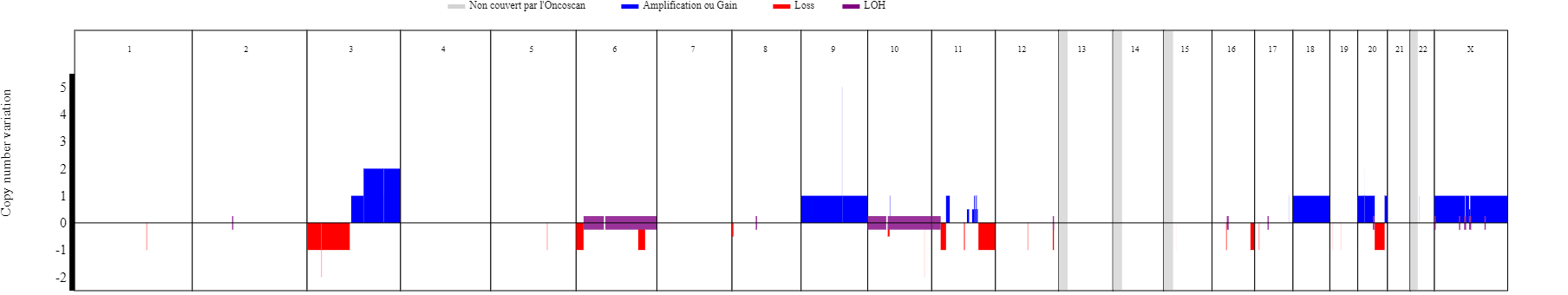


**Alterations concerning whole arms or chromosomes:**

Gain: 3q, 9, 18, 20p. Loss: 3p. LOH: 6q, 10

**Notable alterations**

| Cytoband | Size | Number of copies | Type | Genes of interest |
| --- | --- | --- | --- | --- |
| 3q26.1 | 35162.198 | 4.0 | Gain | SOX2 |
| 11q22.1 | 35962.572 | 1.0 | Loss | ATM |
| 10q11.21 | 93020.982 |  | LOH | PTEN |
| 17q11.2 | 2566.864 |  | LOH | NF1 |

**Clinical case 3**
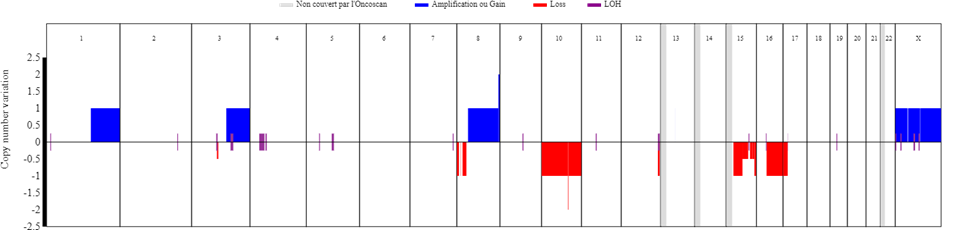


**Alterations concerning whole arms or chromosomes:** Gain: 1q, 8q, Xq, Xp. Loss: 10q, 10p, 15q, 16q.

**Notable alterations**

| Genomic Position | Cytoband |  | Size(Kbp) | | Number of copies | Type | Genes of interest |  |
| --- | --- | --- | --- | --- | --- | --- | --- | --- |
| 3:117'563'472-197'852'564 | 3q13.32 - 3q29 |  | 80'289 | | 3 | Gain | BCL6 |  |
| 3:117'563'472-197'852'564 | 3q13.32 - 3q29 |  | 80'289 | | 3 | Gain | PIK3CA |  |
| 17:400'958-15'565'541 | 17p13.3 - 17p12 |  | 15'164 | | 1 | Perte hétérozygote | TP53 |  |
| 10:89'751'984-91'312'337 | 10q23.31 |  | 1'560 |  | | Perte homozygote (focal) | FAS |  |
| 10:89'578'297-89'689'906 | 10q23.31 |  | 111 |  | | Perte homozygote (focal) | PTEN |  |

**Clinical case 4**
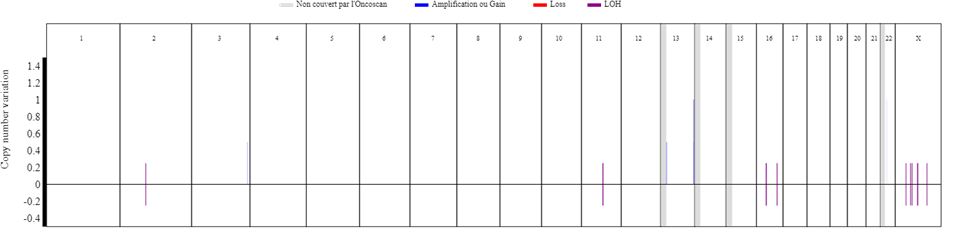


No copy number alterations of arms or entire chromosomes

**Clinical case 5**
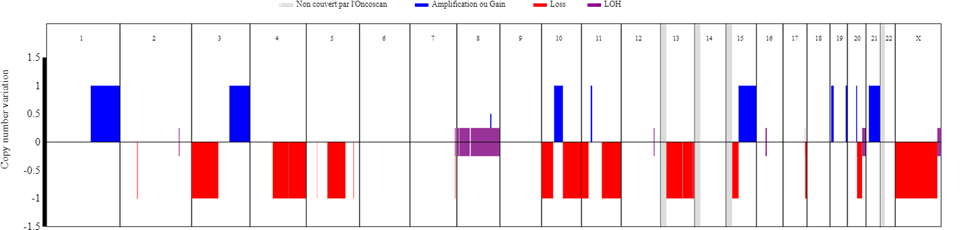


**Alterations concerning whole arms or chromosomes:**

Gain: 1q, 21q. Loss: 8p, 8q. LOH: 3p, 4q, 10p, 11q, 13q, Xq, Xp.

**Notable alterations**

| Genomic Position | Cytoband |  | Size (Kbp) | Number of copies | Type | Genes of interest |
| --- | --- | --- | --- | --- | --- | --- |
| 3:128'161'467-197'852'564 | 3q21.3 - 3q29 |  | 69'691 | 3 | Gain | PIK3CA |

**Clinical case 6**
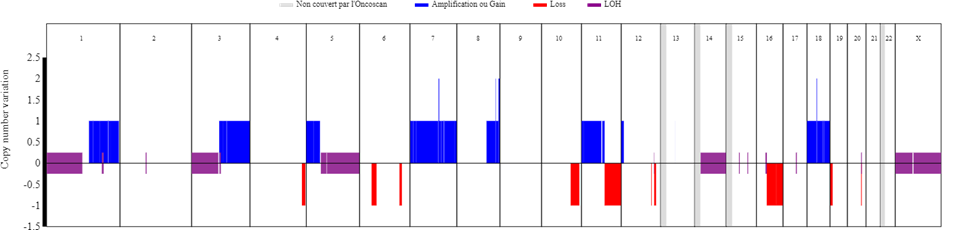


**Alterations concerning whole arms or chromosomes:**

Gain: 1q, 3q, 5p, 7p, 7q, 11p, 18q, 18p LOH: 1p, 3p, 5q, 14q, Xp, Xq Loss: 16q.

**Notable alterations**

| Genomic Position | Cytoband |  | Size(Kbp) | Number of copies | Type | Genes of interest |  |
| --- | --- | --- | --- | --- | --- | --- | --- |
| 3:119'595'644-197'852'564 | 3q13.33 - 3q29 |  | 78'256 | 3 | Gain | BCL6 |  |
| 3:119'595'644-197'852'564 | 3q13.33 - 3q29 |  | 78'256 | 3 | Gain | PIK3CA |  |
|  |  |  |  |  |  |  |  |
|  |  |  |  |  |  |  |  |
|  |  |  |  |  |  |  |  |

**Clinical case 7**
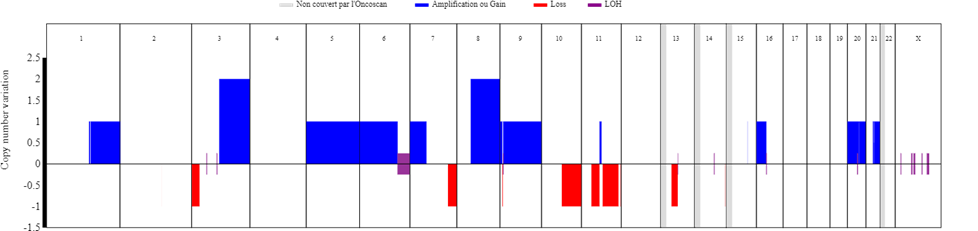


**Alterations concerning whole arms or chromosomes:**

Gain: 1q, 3q, 5p, 5q, 6p, 7p, 8q, 9q, 9p, 16p, 20q, 20p

**Notable alterations**

| Genomic Position | Cytoband |  | Size(Kbp) | Number of copies | Type | Genes of interest |
| --- | --- | --- | --- | --- | --- | --- |
| 3:93'517'442-197'852'564 | 3q11.1 - 3q29 |  | 104'335 | 4 | Gain | PIK3CA |

**Clinical case 8**
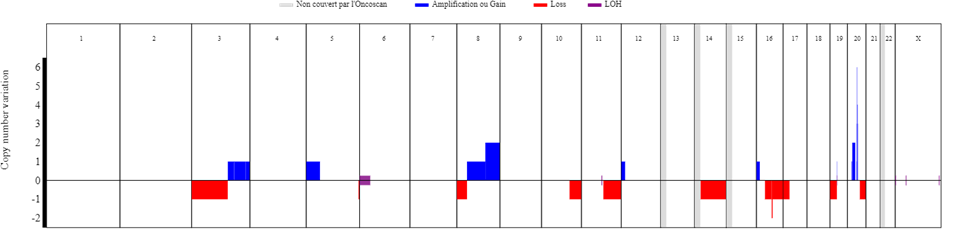


**Alterations concerning whole arms or chromosomes:**

Gain: 5p, 8q. Loss: 3p, 4q, 4p, 10p, 11q, 11p, 14q, 21q

**Notable alterations**

| Genomic Position | Cytoband | Size(Kbp) | Number of copies | Type | Genes of interest |
| --- | --- | --- | --- | --- | --- |
| 20:30'249'779-30'873'322 | 20q11.21 | 623 | 5 | Amplification (focal) | BCL2L1 |
| 3:184'526'207-197'852'564 | 3q27.2 - 3q29 | 13'326 | 3 | Gain | BCL6 |
| 3:144'496'333-183'943'141 | 3q24 - 3q27.1 | 39'446 | 3 | Gain | PIK3CA |
| 17:400'958-21'704'627  16:51'771'623-54'873'802 | 17p13.3 - 17p11.2  16q12.1 - 16q12.2 | 21'303  3'102 | 1 | Perte heterozygote  Perte hétérozygote | TP53 |

**Clinical case 9**
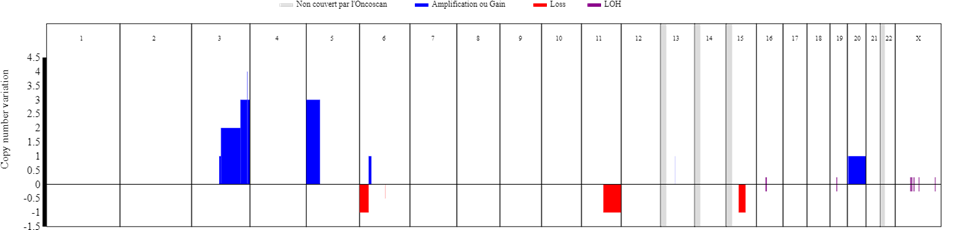


**Alterations concerning whole arms or chromosomes:** Gain: 1q, 8q, Xq, Xp. Loss: 10q, 10p, 15q, 16q.

**Notable alterations**

| Genomic Position | Cytoband |  | Size(Kbp) | | Number of copies | | Type | | Genes of interest | |  |
| --- | --- | --- | --- | --- | --- | --- | --- | --- | --- | --- | --- |
|  |  |  |  |  |  |  |  |  |  |  |  |
| 3:188'402'802-189'665'655 | 3q28 | | | 1'262 | | 6 | | Amplification (focal) | | TP63, LPP |  |
| 3:165'631'380-188'397'584 | 3q26.1 - 3q28 | | | 22'766 | | 5 | | Amplification | | MECOM |  |
| 3:165'631'380-188'397'584 | 3q26.1 - 3q28 | | | 22'766 | | 5 | | Amplification | | PRKCI |  |
| 3:165'631'380-188'397'584 | 3q26.1 - 3q28 | | | 22'766 | | 5 | | Amplification | | SOX2 |  |
| 3:165'631'380-188'397'584 | 3q26.1 - 3q28 | | | 22'766 | | 5 | | Amplification | | BCL6 |  |
| 5:38'138-46'401'271 | 5p15.33 - 5p11 | | | 46'363 | | 5 | | Amplification | | IL7R |  |
| 5:38'138-46'401'271 | 5p15.33 - 5p11 | | | 46'363 | | 5 | | Amplification | | CTNND2 |  |
| 3:165'631'380-188'397'584 | 3q26.1 - 3q28 | | | 22'766 | | 5 | | Amplification | | TBL1XR1 |  |
| 5:38'138-46'401'271 | 5p15.33 - 5p11 | | | 46'363 | | 5 | | Amplification | | TERT |  |
| 5:38'138-46'401'271 | 5p15.33 - 5p11 | | | 46'363 | | 5 | | Amplification | | DROSHA |  |
| 5:38'138-46'401'271 | 5p15.33 - 5p11 | | | 46'363 | | 5 | | Amplification | | RICTOR |  |
| 3:165'631'380-188'397'584 | 3q26.1 - 3q28 | | | 22'766 | | 5 | | Amplification | | PIK3CA |  |
| 20:3'199'768-62'912'463 | 20p13 - 20q13.33 | | | 59'712 | | 3 | | Gain | | BCL2L1 |  |

**Clinical case 10**
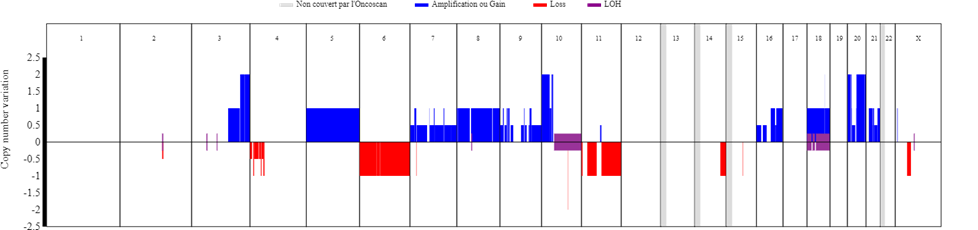


**Alterations concerning whole arms or chromosomes:**

Gain: 5q, 5p, 7q, 7p, 8q, 8p, 9q, 10p, 16q, 16p, 18p, 18q, 20q, 20p, 21q LOH: 10q, 18q, 18p Loss: 4p, 6p, 6q, 11q

**Notable alterations**

| Genomic Position | Cytoband |  | Size(Kbp) | Number of copies | Type | Genes of interest |  |
| --- | --- | --- | --- | --- | --- | --- | --- |
| 3:179'546'343-197'852'564 | 3q26.33 - 3q29 |  | 18'306 | 4 | Gain | BCL6 |  |
| 3:164'654'373-179'228'364  10:89'077'119-89'944'820 | 3q26.1 - 3q26.33  10q23.2 - 10q23.31 |  | 14'573  867 | 4 | Gain  Perte homozygote* | PIK3CA  PTEN |  |
|  |  |  |  |  |  |  |  |
|  |  |  |  |  |  |  |  |
|  |  |  |  |  |  |  |  |
